# Enhancing biological imaging with high-performance probes based on thermally activated delayed fluorescence

**DOI:** 10.1016/j.isci.2025.113526

**Published:** 2025-09-11

**Authors:** Yunfei Xia, Meizhu Zheng, Xue Li, Kai Song, Zhisheng Teng

**Affiliations:** 1 School of Life Science, Changchun Normal University, Changchun 130032, China; 2 Institute of Innovation Science and Technology, Changchun Normal University, Changchun, China; 3 Department of Head and Neck Surgery, Zhejiang Cancer Hospital, Hangzhou, China; 4 Hangzhou Institute of Medicine (HIM), Chinese Academy of Sciences, Hangzhou, China

**Keywords:** Optical imaging, Materials science, Biomaterials

## Abstract

Thermally activated delayed fluorescence (TADF) materials have gained significant attention in biological imaging due to their high quantum efficiency and capacity to suppress short-lived background fluorescence. This review comprehensively analyzes recent advancements in organic TADF probes, focusing on their photophysical mechanisms, structural design, and encapsulation strategies to improve biocompatibility and photostability. We highlight their applications in organelle-targeted imaging, dynamic live-cell tracking, and *in vivo* imaging in model organisms such as zebrafish and mice. Additionally, we discuss current challenges, including oxygen sensitivity and limited long-term stability. The development of high-performance TADF probes promises to enhance imaging quality, facilitate deeper tissue penetration, and support long-term cellular studies, thereby contributing substantially to biomedical research and diagnostic imaging.

## Graphical abstract

**Figure:**
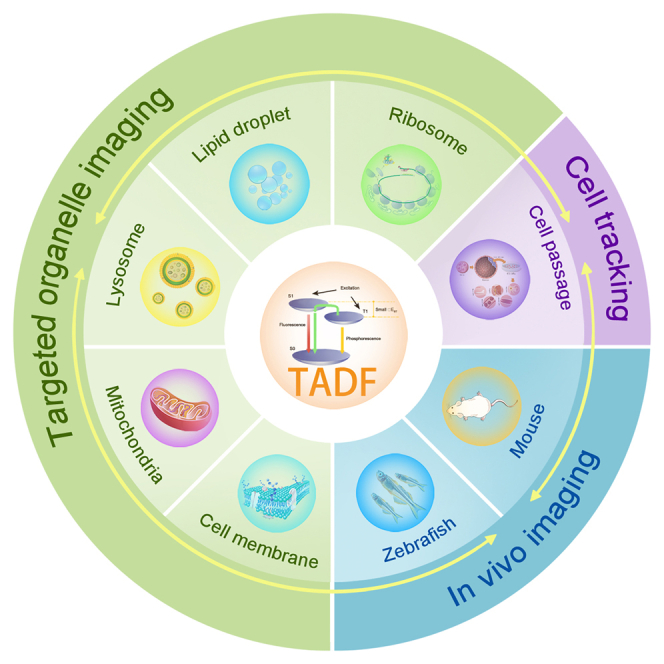

## Introduction

Fluorescence imaging is a noninvasive imaging technique that uses specific fluorescent probes to label particular cells or molecules, enabling the monitoring of various complex physiological activities within living organisms. This technology is of great significance in studying biological mechanisms, early disease detection, accurate diagnosis, and treatment.^1^^,^^2^^,^^3^^,^^4^^,^^5^^,^^6^^,^^7^ Fluorescence imaging, with its high spatiotemporal resolution, high sensitivity, fast real-time monitoring, and simplicity of operation, has become a powerful visualization tool for observing and analyzing molecular activities at the cellular and subcellular levels.^8^^,^^9^^,^^10^ However, due to the complex components of biological tissues, the fluorescence signals generated by traditional fluorescent probes are often interfered with by tissue autofluorescence and light scattering, severely affecting imaging accuracy and signal-to-noise ratio.^11^ Delayed luminescence is a phenomenon in which a material emits light with a significant time delay after cessation of optical excitation, effectively suppressing biological background fluorescence interference.^12^^,^^13^ TADF materials are a special class of materials that offer benefits, such as low cost and the absence of heavy metals. Their luminescence mechanism involves the transition between the excited and ground states via thermal activation, resulting in delayed fluorescence (DF) emission. TADF materials have a sufficiently small energy gap (Δ*E*_ST_) between their singlet-state (S_1_) and triplet-state (T_1_), allowing efficient upconversion from T_1_ to S_1_ through reverse intersystem crossing (RISC), thereby enabling the effective utilization of triplet excitons in photoluminescent systems.^14^ The rational design of the molecular structure can significantly enhance the performance of TADF materials. Therefore, using TADF materials for biological imaging can effectively reduce background fluorescence and light-scattering interference, significantly enhancing the imaging signal-to-noise ratio and sensitivity. However, practical applications require high performance and luminescence efficiency from TADF materials.

**Scheme 1:**
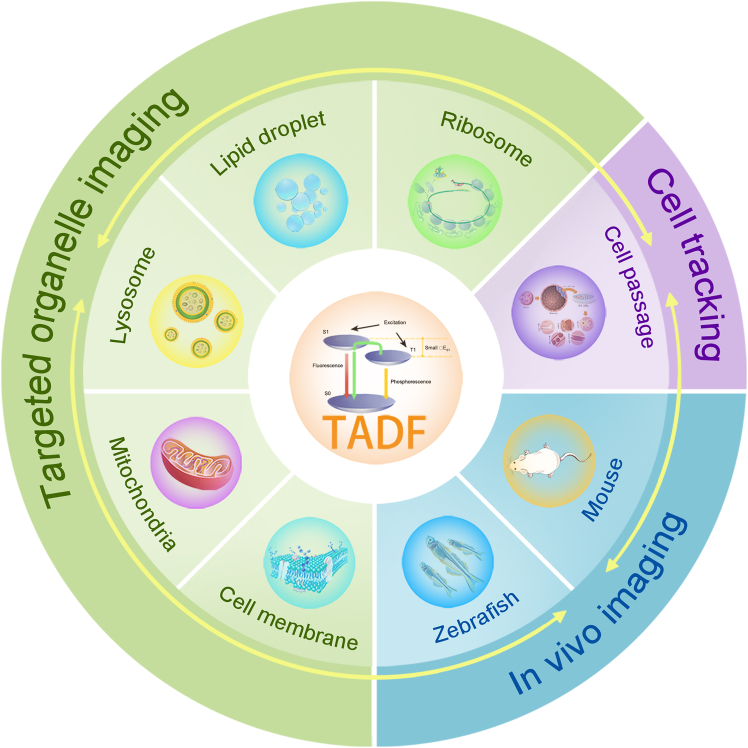
Schematic illustration of TADF materials for different bio-imaging applications

**Figure 1:**
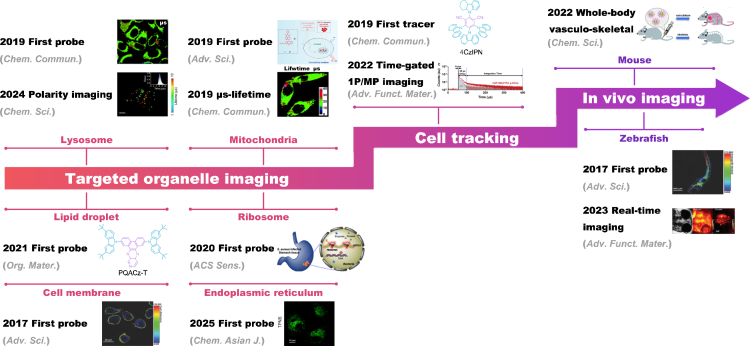
TADF materials reported for targeting organelles, cell tracking, and *in vivo* imaging

Since the significant breakthrough in the study of TADF molecules by Adachi et al. in 2011, a diverse range of TADF materials has been developed.^15^ These organic compounds have garnered extensive attention due to their cost-effectiveness, ease of synthesis, and tunable structural and luminescent properties. Researchers have designed various TADF probes for applications in bioimaging, which not only target specific organelles but also facilitate cell tracking and *in vivo* imaging (Scheme 1). This demonstrates their substantial potential in biomedical research. TADF probes enhance fluorescence imaging signal clarity by facilitating time-resolved detection, which effectively distinguishes specific signals from short-lived background autofluorescence and excitation light scattering. Furthermore, relative to phosphorescent probes, TADF probes achieve long-lived luminescence independently of heavy metals, offering superior biocompatibility and environmental friendliness. However, TADF materials still face challenges such as oxygen quenching effects, and their stability and imaging performance within complex biological environments require further optimization. Currently, improving the biocompatibility of TADF materials and overcoming the quenching of T_1_ by molecular oxygen to maintain long-lived luminescence is crucial. By modifying the structures or selecting suitable encapsulation strategies, highly intense, long-lived, pure organic TADF probes can be obtained, which are significant for fluorescence lifetime imaging (FLIM) and time-resolved luminescence imaging (TRLI).^16^^,^^17^ Although numerous reviews on TADF materials exist, they are predominantly focused on photophysical mechanisms, organic light-emitting diode (OLED) devices, or broad biomedical applications, lacking a systematic discussion dedicated specifically to their application in bioimaging.^18^^,^^19^^,^^20^^,^^21^^,^^22^^,^^23^^,^^24^^,^^25^^,^^26^^,^^27^^,^^28^^,^^29^^,^^30^^,^^31^^,^^32^^,^^33^ For the first time from an application perspective, this review systematically summarizes the design principles, performance optimization strategies, and key breakthroughs of TADF materials in three core bioimaging domains—targeted organelle imaging (e.g., mitochondria and lysosomes), dynamic live-cell tracking, and *in vivo* imaging (zebrafish and mice) —revealing their unique advantages and adaptation mechanisms for cross-scale imaging (Figure 1). Furthermore, addressing critical bottlenecks such as oxygen quenching and poor water solubility hindering the biological application of TADF materials, this work comprehensively reviews multidimensional design strategies including chemical modification, self-assembly nanoengineering, amphiphilic molecular encapsulation, and nanocarrier loading. It systematically evaluates the performance of these strategies in terms of fluorescence lifetime, oxygen quenching resistance, photostability, and biocompatibility. Overall, this study provides a comprehensive overview of TADF materials for bioimaging applications, offers new insights for designing high-performance fluorescent probes, and outlines current challenges alongside future research directions.

## Luminescence mechanism of TADF

**Figure 2:**
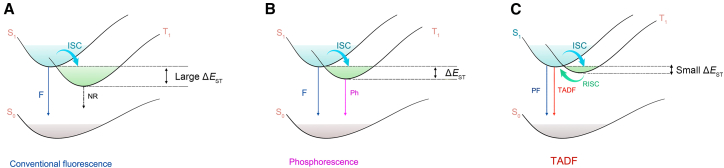
Luminescence mechanism of conventional fluorescence, phosphorescence, and TADF materials (A) Conventional fluorescence process with large ΔE_ST_. (B) Phosphorescence process involving ISC and triplet state emission. (C) TADF mechanism with small ΔE_ST_ enabling RISC. S_0_, ground state; S_1_, lowest singlet excited state; T_1_, lowest triplet excited state; ISC, intersystem crossing; RISC, reverse intersystem crossing; F, fluorescence; Phos, phosphorescence; PF, prompt fluorescence; TADF, thermally activated delayed fluorescence; NR, non-radiative transition.

Organic luminescent materials absorb light, promoting them to excited electronic states. Following photoexcitation in photoluminescent systems, the lowest singlet excited state (S_1_) is typically populated. Some singlet excitons undergo intersystem crossing (ISC) to the triplet state (T_1_). Radiative decay from T_1_ is spin-forbidden, resulting in long-lived emission. In conventional fluorescent materials, emission primarily originates from singlet excitons, as triplet excitons predominantly decay non-radiatively.^34^ However, TADF materials leverage a small singlet-triplet energy gap (Δ*E*_ST_) to facilitate efficient RISC, converting T_1_ back to S_1_ and thereby generating delayed fluorescence. This mechanism not only enhances luminescence efficiency but also imparts long-lived emission lifetimes (typically microseconds to milliseconds), rendering TADF materials particularly suited for time-resolved fluorescence imaging. Utilizing delayed signal acquisition via time-gated detection effectively suppresses the short-lived autofluorescence background (typically 1–10 ns), thereby significantly improving the signal-to-noise ratio (Figure 2A). On the other hand, phosphorescent materials often incorporate heavy metals such as iridium and platinum(II) to enhance spin-orbit coupling, thereby facilitating ISC from the lowest singlet excited state to the lowest triplet excited state. This enables the utilization of triplet excitons via radiative decay (Figure 2B). However, the rare-earth heavy metals employed in phosphorescent materials are typically expensive, toxic, and environmentally detrimental. Consequently, several strategies have been proposed for harvesting triplet excitons using purely organic materials, including triplet-triplet annihilation (TTA), (TADF) channels, hybridized local and charge-transfer (HLCT) states, and TADF.^35^^,^^36^ TADF materials facilitate the upconversion of triplet excitons to singlet excitons through RISC, yielding delayed fluorescence. This mechanism allows for more efficient utilization of photoexcited states without relying on heavy metals and holds significant promise for high-performance fluorescence imaging applications. Crucially, TADF materials can harness both singlet and triplet excitons without requiring precious metals, theoretically achieving 100% exciton utilization efficiency. Moreover, TADF materials, primarily consisting of pure organic molecules, are highly tunable, efficient, and cost-effective. Thus, TADF materials have become a research hotspot in the field of OLEDs and are promising in biological imaging applications.

The photoluminescence of TADF materials comprises prompt fluorescence (PF) and DF. The TADF materials undergo energy-level transitions upon light excitation without changing their spin states. Some singlet excitons directly radiate back to the ground state, emitting PF with lifetimes in the nanosecond range, like traditional fluorescence.^37^ Owing to the small energy gap (Δ*E*_ST_) between S_1_ and T_1_ in TADF materials, some singlet excitons undergo ISC to the T_1_. Then, triplet excitons upconvert back to singlet excitons via RISC, followed by radiative decay to the ground state. Thus, DF is emitted with lifetimes extending from microseconds to milliseconds due to the multiple photophysical processes (Figure 2C). The long-lived emission of TADF probes enables time-gated detection, which isolates the target fluorescence signal from rapidly decaying autofluorescence and scattering, thereby enhancing the signal-to-noise ratio.^38^^,^^39^ The long-lived fluorescence emission properties of TADF materials make them suitable for FLIM and TRLI, thereby enhancing imaging precision and clarity. Long fluorescence lifetimes are crucial for FLIM, facilitating precise measurement of fluorescence decay times. This capability is vital for differentiating distinct molecular environments and enhancing imaging contrast.

**Figure 3:**
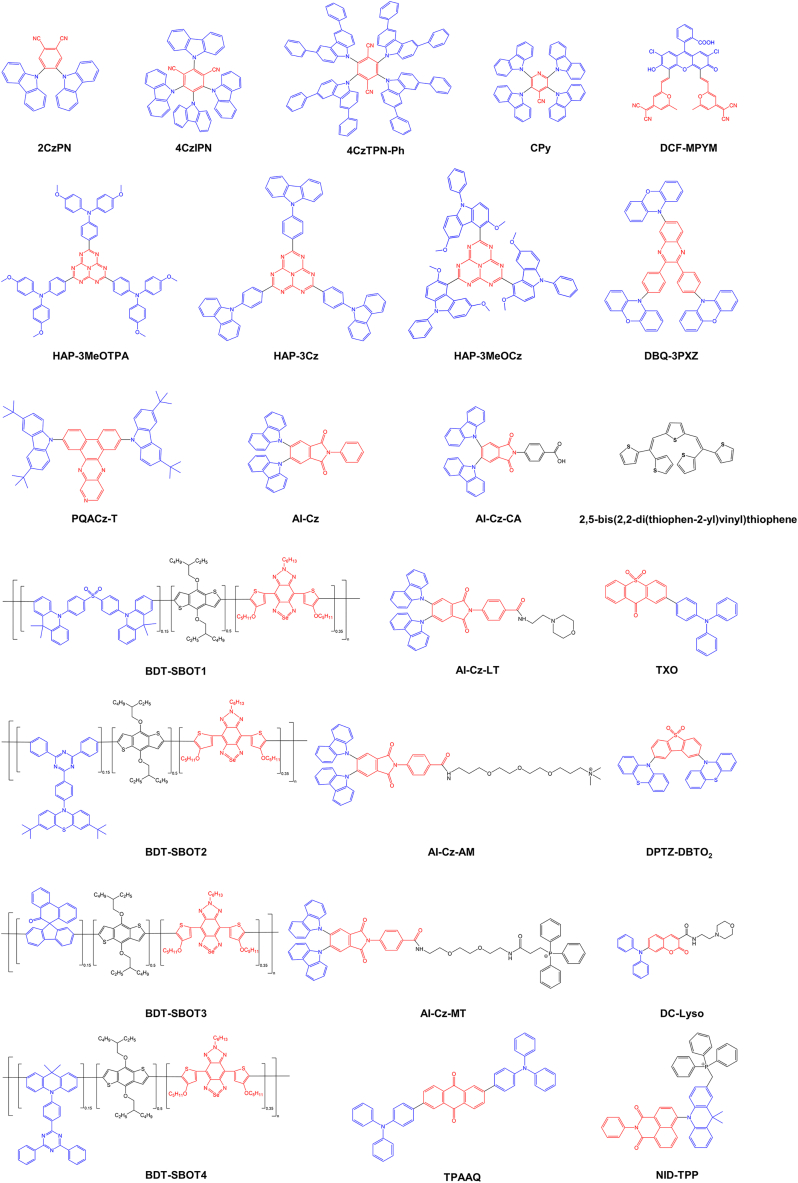
Representative molecular structures of TADF materials designed for bioimaging applications These structures are primarily based on D-A or D-π-A architectures to minimize ΔE_ST_ and promote efficient RISC.

Achieving efficient TADF requires the consideration of various factors, with two core factors being paramount: the Δ*E*_ST_ and photoluminescence quantum yield (PLQY). First, a small Δ*E*_ST_ is crucial for an efficient RISC, which can be achieved by minimizing the orbital overlap between the lowest unoccupied molecular orbital (LUMO) and highest occupied molecular orbital (HOMO) to facilitate effective RISC.^40^ Second, a high PLQY necessitates a large radiative transition rate (k_r_), which is critical for achieving a high PLQY.^41^ According to the Franck-Condon principle, reducing the HOMO-LUMO overlap decreases Δ*E*_ST_; however, this may also reduce k_r_, highlighting a trade-off between lowering Δ*E*_ST_ and increasing PLQY. Therefore, designing TADF materials necessitates comprehensive consideration of Δ*E*_ST_ and PLQY.^42^^,^^43^ Currently, typical TADF molecule structures have been designed and synthesized for diverse bioimaging applications, as indicated in Figure 3. Most TADF molecules are devised based on donor-acceptor (D-A) or donor-π-acceptor (D-π-A) structures. By constructing twisted molecular structures, the overlap between the HOMO and LUMO is reduced, resulting in a smaller Δ*E*_ST_ value.^44^^,^^45^ A smaller Δ*E*_ST_ facilitates ISC between S_1_ and T_1_, allowing triplet excitons to undergo RISC and ultimately producing efficient delayed fluorescence. For example, 4CzIPN is a representative TADF molecule, composed of four carbazole (Cz) donor units and one isophthalonitrile acceptor unit. It exhibits excellent TADF properties and is commonly employed in constructing high-performance fluorescent probes. Another example is the AI-Cz series of molecules. By introducing different targeting groups, selective imaging of mitochondria and lysosomes can be achieved. Combining this targeting capability with their outstanding delayed fluorescence properties, these molecules excel in FLIM.

However, not all TADF molecules possess D-A structures. Some TADF molecules demonstrate DF characteristics, even without D-A structures. For instance, oligothiophene-based molecules display rapid ISC from the singlet excited state to the T_1_ while exhibiting TADF properties. Sarkar et al. designed and synthesized a TADF molecule based on thiophene, specifically oligothiophene compound [2,5-bis(2,2-di(thiophen-2-yl)vinyl)thiophene], which lacked a D-A structure and showed aggregation-induced emission (AIE) enhancement and multi-state-dependent TADF properties.^46^ Furthermore, the rapidly evolving field of multiple resonance thermally activated delayed fluorescence (MR-TADF) materials has recently provided a novel direction for TADF molecular design. MR-TADF materials typically deviate from conventional D-A architectures. Instead, they are based on rigid, planar multiple resonance frameworks, enabling intramolecular spatial separation of the highest occupied molecular orbital (HOMO) and the lowest unoccupied molecular orbital (LUMO), which localize on distinct atoms. This results in exceptionally small Δ*E*_st_, typically less than 0.05 eV, thereby facilitating an efficient RISC process. These materials exhibit not only high efficiency, narrow-bandwidth emission, and high color purity but also possess superior photophysical properties. Consequently, they are widely utilized in OLEDs and are gradually expanding into applications such as bioimaging, demonstrating promising potential.

## Design strategies for TADF probes

TADF materials, known for their long luminescence lifetimes and high fluorescence quantum yields, are widely used in OLEDs. TADF materials are highly suitable for bio-imaging applications owing to their efficient triplet exciton harvesting through reverse intersystem crossing, long emission lifetimes, and heavy-metal-free composition. These characteristics collectively improve the signal-to-background ratio by minimizing autofluorescence interference. However, when used in biological imaging, TADF materials face several challenges and limitations. The most critical issues are improving the biocompatibility of TADF materials and overcoming the quenching of T_1_ by molecular oxygen in polar environments to maintain their high-efficiency luminescence in biological systems.^47^^,^^48^^,^^49^

**Figure 4:**
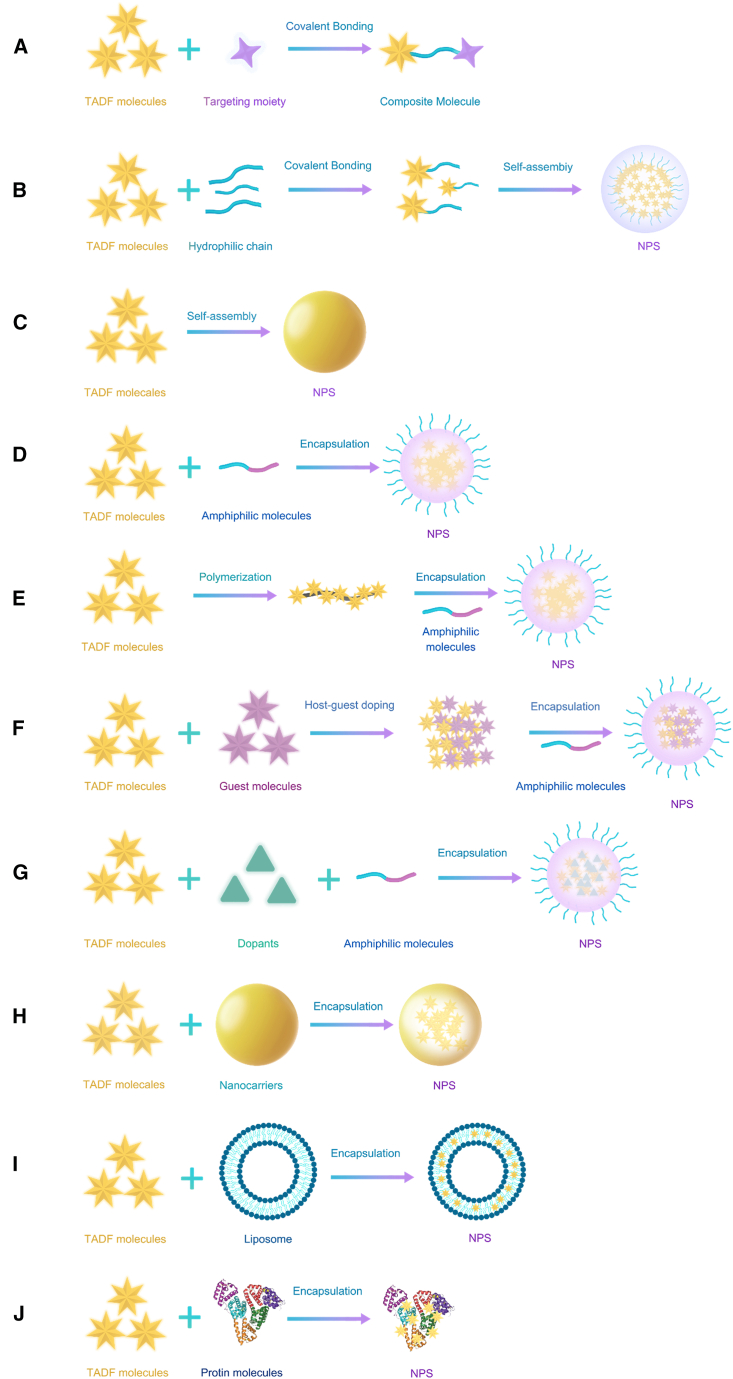
Schematic illustration of design strategies for TADF probes in bio-imaging (A and B) Chemical modification. (C) Self-assembly into nanoparticles. (D–G) Encapsulation using amphiphilic molecules. (H) Nanoparticles as carriers. (I) Liposomes as carriers. (J) Application of biopolymer carriers.

Primarily, TADF molecules exhibit poor water solubility and suboptimal photostability in polar media. Although a few water-soluble TADF materials exist, most are based on twisted D-A structures, which typically display highly hydrophobic rigid structures, making their water solubility negligible. Moreover, the highly polar biological environment can interfere with the internal charge-transfer mechanisms of TADF molecules, affecting the TADF effect and leading to suboptimal luminescence performance. Another significant challenge in biological imaging with TADF materials is the easy quenching of triplet excitons by water and molecular oxygen. Researchers have developed various strategies to improve the biocompatibility of TADF materials and reduce the quenching of triplet states by molecular oxygen (Figure 4). These strategies include chemical modification of TADF molecules; self-assembly into nanoparticles (NPs); and encapsulation using amphiphilic polymers, surfactants, nanocarriers, liposomes, and biomolecules (Table 1).

**Table 1:** Summary of the design strategies, photophysical properties, and targeting of TADF probes discussed in this review

TADF probes	TADF molecules	Design strategies	τTADF; test condition	PLQY	Bio-targeting	Refs.
AI-Cz-NP	AI-Cz	hydrophilic modification and self-assembly	10.68 μs/15 μs; aerated aqueous solution/HepG2 cells	1.36%; water	HepG2 cells	Wei et al.^50^
NID-TPP	NID	targeted modification and AIDF	1.2 μs; sodium tetraphenylborate	0.015%/0.6%; aqueous solution/sodium tetraphenylborate	mitochondria	Ni et al.^51^
AI-Cz-MT	AI-Cz	targeted modification	6.8 μs/16.7 μs; degassed toluene/HepG2 cells	7.3%; 2-MeTHF	mitochondria	Zhang et al.^52^
AI-Cz-LT	AI-Cz	targeted modification	6.3 μs/28.0 μs; degassed toluene/HepG2 cells	8.3%; 2-MeTHF	lysosome	Zhang et al.^52^
AI-Cz-Neo	AI-Cz-CA	targeted modification	17.8 μs/26.7–28.7 μs; PBS in air/S. aureus and E. coli	/	ribosomes	Xu et al.^83^
TPQS	TPQS	targeted modification	13.1 μs;N_2_-purged DMSO	19%; DMSO	endoplasmic reticulum	Shradha et al.^87^
DCF-MPYM	DCF-MPYM	encapsulation with bovine serum albumin (BSA)	22.11 μs/4.3 μs; degassed/aerated ethanol	/	MCF-7 cells	Xiong et al.^63^
AI-Cz-M	AI-Cz	encapsulation with amphiphilic polymers	299 μs/162 μs; aerated aqueous solution/HeLa cells	18.77%; normal saline solution	lipid droplets	Xu et al.^79^
PhDPA-DiKTa g-Odots/MeODPA-DiKTa g-Odots	PhDPA-DiKTa/MeODPA-DiKTa	encapsulation with amphiphilic polymers	203.9 μs/131.6 μs; aqueous solution	77%/38%; aqueous solution	lysosome	Si et al.^75^
4CzIPN organic dots	4CzIPN	encapsulation with amphiphilic copolymers	1.47 μs; aqueous solution	/	HeLa cells	He et al.^54^
BDT-SBOT2 Pdots	T2	monomer polymerization and encapsulation with amphiphilic lipid	0.61 ns; water	1.58%; aqueous solutions	mouse	Hsu et al.^55^
BBSF@CBP NPs	BBSF@CBP	host-guest doping and encapsulation with surfactant	16.17 ns; aqueous solution	0.375%; aqueous solution	HeLa cells	Lv et al.^58^
New type of hybrid fluorescent nanoparticles	DBQ-3PXZ	AIDF, antifading agent, triplet energy barrier, and encapsulation with surfactant	3.2 μs; aqueous solution	3.8%; aqueous solution	4T1 cells	Luo et al.^59^
CPy-Odots	CPy	encapsulation with amphiphilic polymers	9.3 μs; ambient atmosphere	38.3%; Milli-Q water	cell membrane and zebrafish	Li et al.^66^
DCL-NPs	DC-Lyso	solvent evaporation method	1 μs; aqueous solution	10%; aqueous solution	lysosomes	Das et al.^74^
4CzIPN g-Odots	4CzIPN	rigid host matrix and encapsulation with amphiphilic polymer	3.1 μs; air or Ar saturated water	94%; water	HEK293 cells	Tsuchiya et al.^57^
HAP-3MeOTPA g-Odots	HAP-3MeOTPA	rigid host matrix and encapsulation with amphiphilic polymer	67-78 μs; air-saturated aqueous solution or N2-sparged solution	1%; air-saturated aqueous solution or N2-sparged solution	HEK293 cells	Mayder et al.^88^
TXO NPs	TXO	encapsulation with amphiphilic copolymer	4.2 μs; PBS purged with air or N2 or O2	7.42%; PBS	HepG2 cells and zebrafish	Hu et al.^109^
TPAAQ/CBP/CPP NPs	TPAAQ	host-guest assembling and encapsulation with amphiphilic polymer	0.1-0.2 ms; zebrafish	35%; aqueous solution	zebrafish	Zhu et al.^112^

Note: for entries where the “Bio-targeting” column lists a cell line (e.g., HeLa, HepG2, and MCF-7), the probe is considered non-specific to subcellular organelles unless otherwise stated. In these cases, the probes exhibit general cellular uptake without organelle-level targeting.

### Chemical modification

The chemical modification of TADF molecules enhances their biocompatibility and overcomes the quenching of T_1_ by molecular oxygen. Hydrophilic modifications improve their inherent hydrophobicity, increasing their dispersion and stability in polar media (Figure 4A). Hu et al. proposed a self-assembly strategy to eliminate oxygen quenching by modifying the hydrophobic TADF molecule, AI-Cz, with a positively charged hydrophilic chain to synthesize the amphiphilic monomer, AI-Cz-AM.^50^ When dispersed in an aqueous solution, AI-Cz-AM self-assembles into NPs with good biocompatibility and low oxygen sensitivity, called AI-Cz-NP. The positive charge on the NP surface significantly enhances cellular penetration. This self-assembly method effectively overcomes T_1_ quenching by molecular oxygen, resulting in TADF probes with long luminescence lifetimes. Time-resolved fluorescence imaging (TRFI) results showed that after incubation with 10 μM AI-Cz-NP for 2 h, HepG2 cells exhibited an average fluorescence lifetime of approximately 15 μs. Targeted modifications confer specificity, allowing the targeting of specific tissues, cells, or organelles. Further studies have demonstrated the impact of different targeting modifications on the performance of the TADF probes (Figure 4B). Yang et al. synthesized a hydrophilic mitochondria-targeting TADF probe, NID-triphenylphosphonium (TPP^+^) group with TADF properties via non-conjugated methylene.^51^ Similarly, Hu et al. designed two biocompatible, organelle-specific TADF probes, AI-Cz-MT and AI-Cz-LT, by covalently binding the TADF molecule AI-Cz-CA with the mitochondria-targeting TPP and lysosome-targeting 2-morpholinoethylamine groups, respectively.^52^ These probes specifically target mitochondria and lysosomes at a dosing concentration of 10 μM with a 2-h incubation period. Notably, AI-Cz-MT and AI-Cz-LT exhibited high fluorescence quantum yields, long fluorescence lifetimes (6.8 and 6.3 μs) in solution, and long fluorescence lifetimes (16.7 and 28.0 μs) in HepG2 cells during TRFI.

### Self-assembly into NPs

Hydrophobic molecules can self-assemble into aggregates under appropriate conditions through hydrophobic interactions, forming hydrophilic NPs with hydrophilic surfaces (Figure 4C). Lee et al. used three typical TADF molecules, 2CzPN, 4CzIPN, and 4CzTPN-Ph, to self-assemble into well-dispersed nanorods and NPs in deionized water using the nanoprecipitation method.^53^

### Encapsulation using amphiphilic molecules

Amphiphilic molecules, such as amphiphilic polymers and surfactants, self-assemble into micelles in polar solutions, encapsulating hydrophobic TADF molecules within the hydrophobic cavity to effectively isolate oxygen and prevent T_1_ quenching (Figures 4D–4G).

#### Polymer organic dots (a-Odots)

Zhang et al. synthesized a TADF emitter, 4CzIPN, employing carbazole as the electron donor and dicyanobenzene as the electron acceptor.^54^ This molecule exhibits excellent photophysical properties, including a high PLQY, a long delayed fluorescence lifetime (1.47 μs), and notable two-photon absorption (TPA) characteristics. The TPA cross-section (σ_2_) of 4CzIPN in tetrahydrofuran (THF) solution was measured as 9 GM (1 GM = 10^−50^ cm^4^ s photon^−1^) at 800 nm excitation wavelength. Although this σ_2_ is lower than the peak values reported for some standard dyes (e.g., Rhodamine B at optimal wavelengths), the long fluorescence lifetime of 4CzIPN enables effective suppression of short-lived autofluorescence interference in biological tissues. This property, combined with its biocompatibility, enhances its suitability for two-photon FLIM applications, as demonstrated in cellular FLIM experiments. To improve the biocompatibility of 4CzIPN in living cells, researchers used the nanoprecipitation method to assemble an amphiphilic copolymer (PEG-b-PPG-b-PEG) with 4CzIPN in an aqueous solution to form stable 4CzIPN organic dots. Two-photon FLIM results showed that 4CzIPN organic dots were easily absorbed by cells, uniformly distributed in the cytoplasm, and effectively eliminated autofluorescence from molecular components in biological tissues.

#### Polymer dots

Chan et al. first copolymerized TADF monomers with donor and acceptor segments through Stille coupling to synthesize a conjugated polymer containing TADF molecules.^55^ This conjugated polymer was then co-precipitated with amphiphilic lipids to form semiconductor polymer dots (Pdots) with near-infrared II (NIR-II) emission. Amphiphilic lipids with hydrophilic polyethylene glycol segments effectively reduced non-specific biomolecule adsorption, prolonged the blood circulation time of Pdots, enabled effective *in vivo* bone targeting, and allowed visualization of the vascular and skeletal systems of mice at different time points.

Hudson et al. designed orange-/red-emitting Pdots, consisting of hydrophilic guanidine-rich blocks as cell-penetrating peptide mimics and rigid organic semiconductor blocks providing effective TADF.^56^ The rigid carbazole-based matrix, mCPN, used for doping, restricted intramolecular motion and also shielded TADF molecules from oxygen quenching in polar environments. These Pdots efficiently entered HeLa, CHO, and HepG2 cells, localized outside the lysosomes, and maintained high cell viability even at concentrations up to 25 mg/mL.

#### Glassified organic dots

Adachi et al. combined the TADF emitter, 4CzIPN, with the amphiphilic polymer DSPE-PEG2k.^57^ The glassy matrix, 1,3-bis(N-carbazolyl)benzene (mCP), formed rigid spherical NPs with an average diameter of 371 nm, named glassified organic dots (g-Odots). The glass matrix isolates the oxygen environment, allowing the TADF probe to maintain an extended fluorescence lifetime.

#### Host-guest doped NPs

Lv et al. used BBSF as a guest molecule and CBP as a host molecule to synthesize host-guest doped BBSF@CBP microcrystals via reprecipitation.^58^ These BBSF@CBP microcrystals exhibited outstanding amplified stimulated emission and TADF properties, facilitating low-power stimulated emission depletion (STED) imaging by reducing the saturation intensity. Pluronic F-127 was used to encapsulate BBSF@CBP microcrystals into amphiphilic BBSF@CBP NPs to improve their biocompatibility. These NPs achieved a fluorescence quantum yield of 0.375, produced a red emission peak at 642 nm, a Stokes shift of 5,700 cm^−1^, and a lifetime of 16.17 ns, with high photostability, making them suitable for STED imaging.

#### Novel hybrid fluorescent NPs

Tian et al. designed and synthesized a novel hybrid fluorescent NP consisting of aggregation-induced delayed fluorescence (AIDF) dye (DBQ-3PXZ) with significant TADF and AIE properties, an amphiphilic polymer matrix (PSMAc), small-molecule anti-photobleaching agents (singlet oxygen quencher and antioxidant), and high-triplet-energy fragments (Si-mCP).^59^ By shielding oxygen through amphiphilic polymers, suppressing oxygen-mediated photodegradation with anti-photobleaching agents, and enhancing fluorescence with high-triplet-energy fragments, these hybrid NPs achieved an average fluorescence lifetime of 2.7 μs.

### NPs as carriers

Using NPs as carriers to encapsulate TADF molecules can enhance their stability in polar media, maintain effective optical performance, and reduce cytotoxicity (Figure 4H). Dias et al. encapsulated hydrophobic TADF dyes into polystyrene (PS) NPs via swelling methods to create fluorescent probes.^60^ This strategy improves the luminescence performance of TADF dyes by shielding solvent effects, allowing their application in polar media and making them suitable for live-cell imaging. Amino-modified PS particles are more easily internalized by the cells and dispersed uniformly within the cells. Dias et al. also doped TADF dyes into silica NPs or polyethylene glycol-modified silica NPs, avoiding the solvent polarity effect on the dyes and effectively retaining their optical properties in polar media.^61^ These NPs were efficiently internalized by human cells even at low culture concentrations and primarily localized in the cytoplasm, enabling fluorescence live-cell imaging.

### Liposomes as carriers

Liposomes, composed of a phospholipid bilayer with hydrophobic tails and hydrophilic heads, can encapsulate water- and lipid-soluble substances, providing good biocompatibility and stability (Figure 4I). Palsson et al. utilized liposomes as carriers for TADF molecules in live-cell delivery and internalization, avoiding significant oxygen quenching of TADF emission while enhancing cellular uptake and retention of TADF molecules.^62^ Before encapsulating TADF molecules in liposomes, it is necessary to remove molecular oxygen during repeated freeze-thaw steps to maintain structural integrity. Time-resolved fluorescence microscopy initially showed PF, followed by DF emitted by the TADF probes internalized by HepG2 cells.

### Application of biopolymer carriers

Certain biopolymers, such as bovine serum albumin (BSA), can act as delivery carriers to transport TADF molecules into cells, providing a hydrophobic cavity that isolates TADF from oxygen and water, effectively resisting T_1_ quenching (Figure 4J). Song et al. constructed an organic small molecule TADF probe, DCF-MPYM, based on a fluorescein derivative.^63^ DCF-MPYM exhibited significant TADF properties, a large Stokes shift, low cytotoxicity, and a long fluorescence lifetime (up to 22.11 μs in deoxygenated ethanol), demonstrating the potential for live-cell time-resolved imaging. DCF-MPYM displayed oxygen sensitivity and lower fluorescence intensity under aerobic conditions. By incorporating BSA, DCF-MPYM was isolated in the hydrophobic cavity of BSA, which quenched singlet oxygen with tryptophan in BSA, enhancing the DF intensity. Moreover, BSA facilitated the penetration of the probe into cells, improving the biocompatibility of the molecular probe.

### Carbon dot

The luminescence mechanism of TADF necessitates a small energy gap between the singlet (S_1_) and triplet (T_1_) states. Carbon quantum dots (CDs) are engineered to meet this requirement, achieving an energy difference of less than 0.1 eV between these states. Additionally, the embedding of CDs into a substrate effectively suppresses the vibrations and rotations of the luminescent groups, thereby minimizing the incidence of non-radiative transitions. These factors collectively contribute to a reduced energy gap between the singlet and triplet states, facilitating effective TADF luminescence. Jie et al. synthesized CDs via a straightforward one-step hydrothermal method using citric acid, diethanolamine, and boric acid, subsequently embedding them in a B2O3 substrate.^64^ This process resulted in deep-blue luminescence from the TADF phenomenon. The boron-doped carbon dots (BNCDs) demonstrated remarkable stability and achieved a quantum yield of up to 24.1%, making them potentially valuable for bioimaging applications. Meanwhile, Li et al. designed and synthesized multifunctional TADF carbon dots (M-FNCDs) that exhibited stable TADF properties in aqueous solutions. These M-FNCDs also displayed temperature-responsive behavior, effectively serving as temperature sensors over a wide range from 77 K to 370 K.^65^

### Strategies to overcome oxygen sensitivity in TADF materials for bioimaging

In the aforementioned design strategies for TADF probes, various encapsulation strategies play a crucial role in enhancing the biocompatibility and photostability of the materials. However, the superior performance exhibited by these strategies under experimental conditions is often significantly compromised in physiological environments due to interference from molecular oxygen. Specifically, the triplet excited state (T_1_) is highly susceptible to quenching by molecular oxygen, thereby suppressing the emission of delayed fluorescence and causing a substantial decrease in the high PLQY measured experimentally when applied *in vivo*. This severely limits the practical performance of TADF materials in bioimaging applications. Existing research indicates that different encapsulation and modification strategies exhibit markedly distinct capabilities in resisting oxygen quenching. For instance, DCF-MPYM demonstrates a lifetime of 22.11 μs in deoxygenated ethanol but rapidly decreases to 4.3 μs in air, highlighting the high dependence of its TADF characteristics on an oxygen-depleted environment. In contrast, NID-TPP achieves an AIDF effect via mitochondria-induced aggregation, resulting in nearly identical lifetimes under both oxygenated and deoxygenated conditions (0.82 vs. 0.83 μs). Its PLQY also increases from 0.015% in the monomeric state to 0.6% in the aggregated state, demonstrating appreciable oxygen resistance. Within the AI-Cz series, the lifetime of AI-Cz-MT recovers from complete quenching to 12 μs upon addition of BSA in phosphate-buffered saline (PBS). Conversely, AI-Cz-LT loses its TADF property under identical conditions, underscoring the significant influence of molecular structure and modification strategy on oxygen sensitivity. Self-assembly strategies also exhibit excellent performance. AI-Cz-NP achieves a lifetime of 10.68 μs and a PLQY of 1.36% in air-saturated water and maintains delayed luminescence of approximately 15 μs within cells. This performance is markedly superior to that of the monomeric AI-Cz-AM, which is completely quenched in air. Glassy encapsulation strategies, exemplified by 4CzIPN g-Odots, are particularly outstanding. These dots exhibit identical lifetimes of 3.1 μs under both air and argon atmospheres, coupled with a remarkably high PLQY of 94%. This exceptional performance is attributed to the effective barrier against oxygen diffusion provided by the rigid glassy matrix, making this one of the most effective current strategies for mitigating oxygen quenching. Pdots, such as BDT-SBOT2, also demonstrate good oxygen resistance, maintaining a PLQY of 1.58% in both air and inert atmospheres, rendering them suitable for deep-tissue *in vivo* imaging. In summary, glassy encapsulation (g-Odots) and self-assembled nanostructures following hydrophilic modification (AI-Cz-NP) are the most effective strategies for preserving TADF characteristics under physiological conditions. These approaches provide crucial assurance for the application of TADF probes in high signal-to-noise ratio, long-lifetime bioimaging. Collectively, these strategies highlight the performance challenges encountered when transitioning from deoxygenated experimental settings to oxygen-rich physiological environments and underscore the necessity of constructing physical barriers to impede oxygen diffusion for realizing the biological applications of TADF materials.

While physical barriers (e.g., glassy matrices and self-assembled structures) effectively isolate TADF emitters from molecular oxygen to preserve T_1_ and extend delayed fluorescence lifetimes, this isolation concurrently attenuates phototoxic risks. Phototoxicity primarily arises from energy transfer of T_1_ to ambient oxygen, generating cytotoxic reactive oxygen species (ROS). This risk escalates under high-intensity or prolonged illumination, particularly within the high-energy UV-blue spectral range (350–488 nm), where elevated photon energy directly damages biomolecules (e.g., inducing DNA breaks) and amplifies ROS yields. For instance, TADF probes excited by blue light frequently exhibit significant phototoxicity, driven largely by high singlet oxygen quantum yields (ΦΔ). In contrast, probes operating in the NIR-II window typically demonstrate substantially improved cell viability, even at comparable or reduced irradiance. This advantage stems from the inherently lower photon energy and superior tissue penetration depth of NIR-II light, collectively mitigating photodamage. Thus, oxygen-shielding strategies not only enhance luminescent stability but also suppress ROS-mediated phototoxicity. Future emitter designs should prioritize NIR excitation (>600 nm) combined with robust oxygen barriers (e.g., the mCP matrix in g-ODots) to concurrently address oxygen quenching and phototoxic effects.

### Comparative evaluation of encapsulation strategies

Despite continuous advancements in encapsulation strategies for TADF probes, systematic comparison of diverse methodologies remains crucial for informed selection tailored to specific biological applications (Table 2). The key advantages and limitations of seven common strategy classes are summarized below.

**Table 2:** Comparative analysis of TADF encapsulation strategies in terms of cytotoxicity, scalability, photostability, and oxygen quenching resistance

Encapsulation strategy	Cytotoxicity	Scalability	Photostability	O_2_ quenching resistance
Chemical modification	low	high	medium	high
Self-assembly into NPs	high	high	high	high
Amphiphilic molecule encapsulation	low	medium	high	medium/high (with doping)
NPs as carriers	low	high	medium/high	medium (requires oxygen removal)
Liposomes as carriers	low	high	medium	medium (encapsulation improves)
Biopolymer carriers	low	high	medium	low (needs BSA for enhancement)
Carbon dot	medium	high	medium	high

Chemical modification enables precise endowing of TADF molecules with targeting capability and structural functionality (e.g., AI-Cz-MT and NID-TPP), demonstrating excellent selectivity and long-lived emission (up to 28 μs) in subcellular imaging. However, it typically involves multi-step syntheses and complex processes. Despite these challenges, chemical modification strategies exhibit high scalability (as per table rating). Photostability can be variable and dependent on structural modifications. Amphiphilic molecule self-assembly strategies (e.g., AI-Cz-NP) offer facile synthesis, good biocompatibility, and outstanding resistance to oxygen quenching, sustaining cellular lifetimes of approximately 15 μs. These self-assembled structures also demonstrate high photostability (as per table rating), although they generally exhibit low photoluminescence quantum yields (PLQYs; <2%) and high environmental sensitivity, limiting their utility in complex physiological environments.

Amphiphilic molecule encapsulation encompasses several subclasses: organic dots (O-dots; e.g., 4CzIPN O-dots) achieve a favorable balance between biocompatibility and process feasibility, with moderate-to-high PLQYs and microsecond-scale lifetimes suitable for two-photon FLIM, though they retain potential oxygen sensitivity. Glass-encapsulated organic dots (g-O-dots) leverage rigid mCP matrices to attain ultra-high PLQYs (up to 94%) and excellent photostability (LT_50_ ∼360 min), enabling persistent fluorescence signals in cells for up to 21 days, which makes them ideal for long-term labeling. However, their larger particle size and requirement for oxygen-free preparation constrain clinical applicability.

Host-guest doped NPs (e.g., BBSF@CBP) and hybrid fluorescent NPs (e.g., DBQ-3PXZ) optimize oxygen barrier properties and exciton stabilization through structural design, achieving PLQYs up to 37.5% and enabling STED microscopy. This strategy, however, involves complex multi-component doping and interfacial control. NP carrier encapsulation methods (e.g., PS3/PS4) confer microsecond-scale lifetimes (up to 11.8 μs) and moderate PLQYs (0.24) via dye doping, alongside favorable intracellular distribution and imaging performance. Despite providing partial oxygen resistance, deoxygenation is often necessary to maintain lifetime and emission efficiency. Liposome carrier encapsulation effectively encapsulates hydrophobic TADF dyes (e.g., DPTZ-DBTO2), facilitating cytoplasmic localization and microsecond-scale lifetimes (e.g., τ_3_ = 62.3 μs). However, the delayed fluorescence remains highly oxygen-sensitive, exhibiting full performance only under deoxygenated conditions, and stability in biological environments requires further improvement.

Biomolecule carrier encapsulation (e.g., BSA-encapsulated DCF-MPYM) utilizes protein networks to shield oxygen, enhancing delayed fluorescence lifetimes (4.3–15.7 μs) and offering good biocompatibility and solubility for cellular imaging and protein target recognition. Its strong dependence on specific proteins, however, limits general applicability. Finally, the carbon dot (CD) strategy (e.g., BNCDs and M-FNCDs) provides ultra-long lifetimes (up to 2.02 s), favorable scalability, and environmental stability. M-FNCDs exhibit exceptional resistance to oxygen quenching and possess temperature-/humidity-sensing capabilities. Precise control over CD size, targeting ability, and lifetime modulation nonetheless requires further refinement for high-resolution or dynamic imaging applications.

Regarding biocompatibility, encapsulation strategies significantly enhance the safety and efficacy of TADF probes. Chemical modifications incorporating moieties like TPP^+^ or morpholinoethylamine improve aqueous solubility and minimize nonspecific binding, reducing potential toxicity of exogenous components. Self-assembled NPs (e.g., AI-Cz-NP) demonstrate excellent biocompatibility, with >90% cell viability observed even at 50 μM, although solvent residues and illumination-induced toxicity remain concerns. Amphiphilic systems, including liposomes and polymer dots, also achieve good biocompatibility by reducing protein adsorption and prolonging circulation, ensuring safety in cellular and animal models.

Rating criteria applied consistently across the strategies (Table 2) were based on cytotoxicity, scalability, photostability, and oxygen quenching resistance, with low/medium/high ratings reflecting experimental data. Cytotoxicity was rated “low” for cell viability exceeding 90% at imaging concentrations, “medium” for viability between 80% and 90%, and “high” for viability below 80% or upon significant dose-dependent toxicity. Scalability was rated “high” for simple, scalable methods, “medium” for lab-scale production, and “low” for complex synthesis routes. Photostability was rated “high” for minimal fluorescence intensity loss under UV irradiation, “medium” for indirect evidence of stability, and “low” for insufficient data. Oxygen quenching resistance was rated “high” when the difference in PLQY or lifetime under air versus inert conditions was less than 30%, “medium” for partial oxygen protection, and “low” for severe quenching under aerobic conditions.

In summary, each encapsulation strategy offers distinct advantages in photophysical properties, biocompatibility, scalability, and oxygen quenching resistance. Optimal strategy selection requires careful consideration of the specific application context: rigid g-O-dots are preferred for high stability and long-term tracking, Pdots are suitable for deep-tissue NIR-II imaging, and surface-functionalized NPs enable precise subcellular localization.

## Applications

### Targeted organelle imaging

#### Cell membrane

The cell membrane is composed of lipids, proteins, and carbohydrates. It is a vital organelle that structurally defines the boundary of the cell and provides a stable internal environment. The cell membrane plays a significant role in material transport, energy conversion, and information transmission between the cell and its environment. Monitoring dynamic changes in the cell membrane is critical for understanding cellular physiological states, environmental adaptability, and disease diagnosis and treatment.

**Figure 5:**
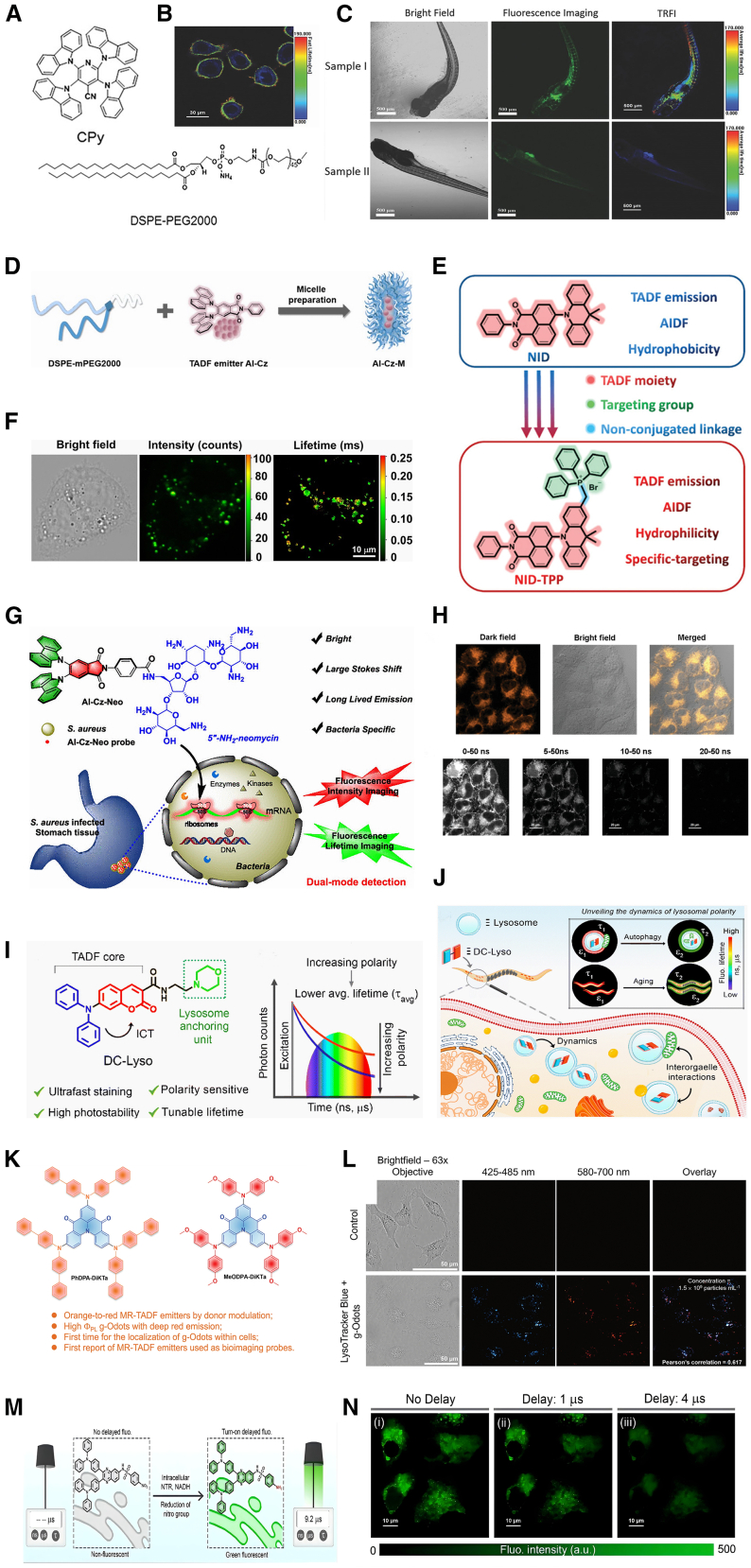
TADF materials for targeted organelle imaging (A) Chemical structures of CPy and DSPE-PEG2000. (B) Fluorescence lifetime image of HeLa cells incubated with CPy Odots for 2 h. (C) Confocal fluorescence images of zebrafish. Sample I/II: injected/non-injected with CPy-Odots. (A–C) Reproduced with permission from Huang et al.^66^ Copyright 2017, Wiley. (D) Schematic illustration of the preparation of the LD-targeting TADF micelle nanoprobe AI-Cz-M. Reproduced with permission from Hu et al.^79^ Copyright 2022, Royal Society of Chemistry. (E) The design of the probe NID-TPP for TRLI. Reproduced with permission from Yang et al.^51^ Copyright 2019, Wiley. (F) FLIM imaging of LDs in HeLa cells incubated with AI-Cz-M for 2 h. Reproduced with permission from Hu et al.^79^ Copyright 2022, Royal Society of Chemistry. (G) Schematic illustration of TADF probe AI-Cz-Neo for dual-mode detection of bacterial 16S rRNA in cells and tissues. Reproduced with permission from Hu et al.^83^ Copyright 2020, American Chemical Society. (H) Steady-state (up) and time-gated luminescence images (down) of HeLa cells after 2 h of incubation with NID-TPP. Reproduced with permission from Yang et al.^51^ Copyright 2019, Wiley. (I) Design of probe DC-Lyso and its relative variation of average fluorescence lifetime with solvent polarity (from left to right). (J) Unveiling autophagy and aging through TRLI of lysosomal polarity with DC-Lyso. (I and J) Reproduced with permission from Patra et al.^74^ Copyright 2023, Royal Society of Chemistry. (K) The chemical structures of compound PhDPA-DiKTa and MeODPA-DiKTa; bullet points present the highlights of this work. (L) HeLa cells co-incubated for 2 h with PhDPA-DiKTa g-Odots and LysoTracker Blue DND-22 imaged with a 63x objective lens (*λ*exc = 405 and 561 nm for LysoTracker and g-Odots, respectively). (K and L) Reproduced with permission from Si et al.^75^ Copyright 2024, Wiley. (M) Schematic representation of turn-on delayed fluorescence response of TPNS in the presence of intracellular nitroreductase (NTR) and NADH, providing dual-mode detection of NTR through luminescence and time-resolved imaging. (N) Time-resolved imaging of HeLa cells incubated with TPNS (5 μM) at the microsecond time domain. Images captured with (i) no time delay and with delay times of (ii) 1 μs and (iii) 4 μs (λex = 405 nm, λem = 488–800 nm); scale bars, 10 μm. The corresponding fluorescence intensity scale is shown. (M and N) Reproduced with permission from Patra et al.^87^ Copyright 2025, Wiley.

Huang et al. encapsulated TADF organic small molecule aggregates of 2,3,5,6-tetracarbazole-4-cyano-pyridine (CPy) in an amphiphilic polymer matrix, DSPE-PEG2000, creating organic quantum dots, CPy-Odots, that specifically label cell membranes (Figure 5A).^66^ These CPy-Odots exhibited excellent water solubility, a fluorescence lifetime of up to 9.3 μs, an absolute fluorescence quantum efficiency of 38.3%, good photostability, low cytotoxicity, and outstanding biocompatibility. In FLIM experiments with HeLa cells (incubated at 500 nM for 2 h), CPy-Odots demonstrated the ability to dynamically track cell membranes, with an average fluorescence lifetime of 165 ns. Notably, CPy-Odots specifically labeled cell membranes without bioconjugation modifications and showed better membrane retention stability than commercial membrane markers such as DiIC18, making them suitable for long-term tracking (Figure 5B). Additionally, CPy-Odots can serve as bright microvascular contrast agents for *in vivo* TRFI in zebrafish after intraventricular injection (300 nL of 0.1 mg/mL solution), thereby providing high-contrast vascular imaging (Figure 5C).

#### Mitochondria

Mitochondria are essential organelles in eukaryotic cells that serve as the primary site of cellular energy metabolism and participate in various vital activities such as Ca^2+^ homeostasis, reactive oxygen species production and clearance, and apoptosis.^67^^,^^68^^,^^69^ Studying mitochondrial morphology and dynamics is important. However, traditional mitochondrial dyes often suffer from aggregation-caused quenching and poor photostability, which affect their imaging performance. In contrast, materials with AIE exhibit enhanced fluorescence upon aggregation and good photostability.

Yang et al. proposed a mitochondria-induced aggregation TRLI strategy by designing and utilizing hydrophilic TADF emitters.^51^ They constructed a hydrophilic, red-emitting, mitochondria-targeting TADF emitter, NID-TPP, by linking the TADF-emitting group NID with TPP^+^ via a non-conjugated methylene (Figure 5E). At a concentration of 10 μM and with a 2-h incubation, significant luminescence was observed in the TRLI of live HeLa cells with a delay time of over 10 ns. Moreover, two-photon luminescence imaging clearly demonstrated the aggregation-induced delayed fluorescence enhancement (AIDFE) phenomenon of NID-TPP in HeLa cells (Figure 5H). This is the first study to report TADF-based live-cell mitochondrial TRLI and two-photon imaging.

#### Lysosomes

Lysosomes are vesicular organelles that contain various acidic hydrolases that perform cellular digestion. The lysosomal microenvironment, including factors like polarity, viscosity, temperature, and pH, is crucial in maintaining normal cellular proliferation, differentiation, metabolism, and function. During lysosome-related physiological processes such as autophagy, lysosomal polarity often changes, and abnormal polarity changes can lead to diseases.^70^^,^^71^^,^^72^^,^^73^ Therefore, quantitative visualization of the lysosomal microenvironment is essential.

Patra et al. developed a TADF probe, DC-Lyso, based on a morpholine-functionalized coumarin-diphenylamine with a D-A structure.^74^ This probe specifically targets lysosomes and features low cytotoxicity and phototoxicity, a large Stokes shift, a polarity-sensitive long lifetime, high photostability, and cellular compatibility, allowing rapid lysosome labeling within 1 min of incubation at a concentration of 10 μM (Figure 5I). DC-Lyso remains in lysosomes for several days after a single incubation, potentially aiding in monitoring lysosomal function during physiological processes, such as cell division and migration. In experiments, water-dispersible nanoaggregates, DCL-NPs, were prepared in a 90% water-dimethyl sulfoxide binary solvent mixture, effectively preventing triplet quenching by oxygen and enhancing the practicality of time-resolved imaging. DCL-NPs exhibited average fast and delayed decay times of 15 ns and 20 μs, respectively. FLIM images of DCL-NP-stained cells and *Caenorhabditis elegans* showed lifetime signals in the microsecond range, indicating the diverse polarity of lysosomes. The polarity changes of lysosomes can reveal complex biological processes such as autophagy and aging (Figure 5J).

Si et al. pioneered the application of MR-TADF materials for bioimaging by designing and synthesizing two orange-red MR-TADF emitters based on a DiKTa core structure: PhDPA-DiKTa and MeODPA-DiKTa.^75^ These emitters were co-encapsulated with mCP within amphiphilic DSPE-PEG_2k_ polymer matrices to form g-Odots (Figure 5K). The resulting g-Odots exhibited excellent aqueous dispersibility and demonstrated significant delayed fluorescence (with τd values of 203.9 μs and 131.6 μs, respectively), enabling single-photon imaging in HeLa cells after 2 h incubation at a concentration of 1.5 × 10^9^ particles mL^−1^. Colocalization experiments revealed a high degree of spatial overlap between the red-fluorescent PhDPA-DiKTa g-Odots and LysoTracker Blue, confirming their specific localization within lysosomes (Figure 5L). This study provides the first experimental validation of the feasibility of MR-TADF materials for bioimaging, reveals their subcellular distribution characteristics, and offers a valuable reference for achieving high signal-to-noise ratio organelle imaging.

#### Lipid droplets

Lipid droplets (LDs) are complex, dynamic organelles that serve as the primary storage sites for neutral lipids; interact with other organelles; and participate in membrane formation and transport, fatty acid transport, protein degradation, inflammation, and viral replication. Abnormal LD dynamics are closely related to various diseases and are important markers for these diseases.^76^^,^^77^^,^^78^

Hu et al. used the amphiphilic polymer, DSPE-mPEG2000, to encapsulate the benzylamide TADF organic small molecule, AI-Cz, creating a TADF nanoprobe, AI-Cz-M, that targets explicitly LDs (Figure 5D).^79^ AI-Cz-M demonstrated good dispersion in aqueous solution, low cytotoxicity, high structural stability, and high cell membrane permeability. When applied at a concentration of 10 μM with 2 h of incubation, this probe showed high selectivity for lipid droplets, enabling real-time, quantitative observation of LD formation and dynamics in live tumor cells and adipocytes. The DSPE-mPEG2000 encapsulation rendered the TADF emission of AI-Cz-M nearly insensitive to oxygen, with a DF lifetime of up to 299 μs in an oxygenated aqueous solution. AI-Cz-M serves as a fluorescent probe to monitor LD accumulation in live cancer cells, displaying a long fluorescence lifetime in tumor cells, with an average of 162 μs (Figure 5F). This was the first instance of an organic TADF probe attaining a fluorescence lifetime in the hundreds of microseconds range for TRFI with high specificity for LDs. Additionally, AI-Cz-M allows real-time quantitative tracking of LD formation and changes during 3T3-L1 preadipocyte differentiation into mature adipocytes.

#### Ribosomes

Ribosomes are supramolecular complexes composed of proteins and RNA, responsible for translating genetic codes on mRNA into proteins that are essential for cell survival and growth. Ribosome fluorescence imaging technology aids in studying ribosome morphology, providing insights into protein synthesis, cellular metabolism, and disease-related biological processes.^80^^,^^81^^,^^82^

Bacterial growth and reproduction depend heavily on ribosomes, rendering ribosome inhibition a common antibacterial strategy. Several antibacterial drugs inhibit bacterial growth by interfering with ribosomal protein synthesis. Hu et al. successfully constructed a hydrophilic TADF probe, AI-Cz-Neo, by conjugating the aromatic acylimine TADF material, AI-Cz-CA, with the aminoglycoside antibiotic neomycin, which specifically targets 16S rRNA (Figure 5G).^83^ This probe can be used for dual-mode detection of bacterial pathogens in cells and tissues at a concentration of 10 μM using confocal laser scanning microscopy (CLSM) and FLIM microscopy. AI-Cz-Neo exhibits strong fluorescence emission, with a maximum emission wavelength of 545 nm. Fluorescence titration experiments showed that fluorescence intensity gradually increased with increasing RNA concentration. Due to specific binding and encapsulation in neomycin, AI-Cz-Neo showed long-lived DF in oxygenated environments. A 1:1 complex of AI-Cz-Neo and A-site oligonucleotide in PBS demonstrated a DF lifetime of 17.8 μs. CLSM and FLIM results of AI-Cz-Neo-stained (10 μM, 2 h incubation) mammalian 264.7 macrophages and mouse gastric tissue slices infected with *Staphylococcus aureus* or *Escherichia coli* MG1655 (10 μM, 4 h incubation) clearly distinguished bacteria from host cells and exhibited long DF lifetimes (26.7–28.7 μs), eliminating short-lifetime tissue autofluorescence interference and significantly improving the signal-to-noise ratio. Hu et al. enabled the specific detection of broad-spectrum bacterial pathogen infections and expanded the potential applications of TADF materials in time-resolved biological imaging.

#### Endoplasmic reticulum

The endoplasmic reticulum (ER) is a key organelle responsible for protein folding, modification, and calcium homeostasis regulation. Dysregulation of its function is closely associated with the pathogenesis of various diseases, including cancer. Molecular probes specifically targeting the ER can dynamically reflect microenvironmental changes, such as aberrant enzymatic activity, providing a crucial molecular foundation for the early diagnosis of related diseases and mechanistic investigations.^84^^,^^85^^,^^86^

Patra et al. designed and constructed a TADF probe, TPQS, based on a triphenylamine (donor)-quinoxaline (acceptor) architecture.^87^ By incorporating a nitrobenzenesulfonamide unit into its structure, they synthesized a “turn-on” derivative, TPNS. This probe possesses ER-targeting capability and undergoes selective nitro group reduction catalyzed by nitroreductase (NTR), triggering a significant “turn-on” delayed fluorescence signal. For cellular imaging, TPNS was applied at a concentration of 5 μM with a 30-min incubation period (Figure 5M). TPNS efficiently responds to endogenous NTR with NADH assistance, enabling highly sensitive and specific detection of NTR activity in cancer cells using both CLSM and TRFI modalities. The D-A structure of TPQS, composed of triphenylamine and quinoxaline, confers characteristic TADF properties: a small Δ*E*_ST_ of 0.17 eV, an emission maximum at 605 nm in dimethyl sulfoxide (DMSO), a quantum yield of 19%, and a delayed fluorescence lifetime of 13.1 μs, demonstrating excellent ISC and RISC capabilities. In HeLa cells, TPQS exhibited rapid ER localization at 5 μM within 5 min of incubation. Its derivative, TPNS, incorporates a p-nitrobenzenesulfonamide group. In the unprocessed state, its fluorescence is quenched via a deactivatable photoinduced electron transfer (d-PET) effect. Upon reduction by NTR/NADH, it is converted to an amino structure, activating “turn-on” delayed fluorescence with an emission maximum at 530 nm and a lifetime of 11.5 μs. In HeLa cells, TPNS localized to the ER (Pearson’s coefficient >0.9 with ER-Tracker) and generated a green fluorescent signal in response to NTR activity. Time-resolved imaging detected long-lived emission (∼9.2 μs), effectively eliminating background autofluorescence interference and achieving high signal-to-noise ratio cellular imaging (Figure 5N). This study reports the first construction of a TADF probe, TPNS, with ER-targeting capability. Utilizing an NTR-triggered delayed fluorescence turn-on mechanism, it enables highly sensitive dual-modal detection of a cancer biomarker, significantly enhancing the precision and application potential of time-resolved imaging within complex biological systems.

### Live cell tracking

Multicellular organisms maintain their normal life activities via the coordination and cooperation of various cells in different organs and tissues. Cells continuously migrate and differentiate from embryonic development onward. Therefore, long-term, non-invasive cell tracking is crucial for understanding various life activities within an organism, such as the origin of specific cell types, organ and tissue development, and the occurrence and progression of diseases.

**Figure 6:**
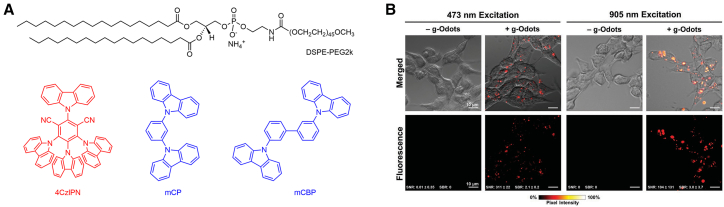
TADF materials for cell tracking (A) Chemical structures of materials for glassy O-dots preparation.^57^ Reproduced with permission from Adachi et al. Copyright 2019, Royal Society of Chemistry. (B) HEK293 cells were incubated with HAP-3MeOTPA g-Odots (+g-Odots) and imaged in both single-photon excitation (λexc = 473 nm) and multiphoton excitation (λexc = 905 nm) modes. Reproduced with permission from Hudson et al.^88^ Copyright 2022, Wiley.

Adachi et al. combined the TADF emitter, 4CzIPN, with the amphiphilic polymer, DSPE-PEG2k, into the glassy matrix, 1,3-bis(N-carbazolyl)benzene (mCP) to prepare glassy organic NPs (Figure 6A).^57^ The g-Odots exhibited good dispersibility, high photostability, high PLQY (94%), and a long-delayed emission lifetime (3.1 μs). For cellular uptake, HEK293 cells were incubated with g-Odots at a concentration of 1.0 mg of freeze-dried particles per 5 mL culture medium for 12 h. Further experiments demonstrated that g-Odots were taken up by cells via micropinocytosis and retained green fluorescence in cells for up to 21 days (10 passages), confirming their utility for long-term cell tracking despite gradual signal attenuation. Hudson et al., based on Adachi’s encapsulation strategy for TADF materials, synthesized three novel s-heptazine TADF materials with green to deep red emissions (λ_max_ = 525–664 nm) and high PLQYs through pseudo-electrophilic substitution or Negishi coupling.^88^ Among them, HAP-3MeOTPA demonstrated superior nonlinear optical properties, strong two-photon absorption, high quantum yield, and photostability, making it suitable for NP preparation for bioimaging experiments. To enhance biocompatibility and dispersibility in aqueous media, TADF materials were encapsulated in a rigid, glassy matrix of mCP with DSPE-PEG2k as the surfactant, resulting in water-dispersible NPs (g-Odots). The HAP-3MeOTPA g-Odots exhibited long-lived DF, orange-red emission, and high PLQY. For cellular imaging, HEK293 cells were incubated with HAP-3MeOTPA g-Odots at a concentration of 1.63×10^9^ particles/mL for 24 h. Cellular experiments indicated that HAP-3MeOTPA g-Odots displayed noticeable fluorescence attenuation after one to two cell passages compared to the previously reported 4CzIPN g-Odots. This could be due to the smaller diameter of HAP-3MeOTPA g-Odots, affecting their retention time within cells (Figure 6B). Furthermore, adjusting the particle diameter may extend the retention time of g-Odots within the cells.

The g-Odots are rigid spherical NPs, with the glassy and rigid host matrix accounting for approximately 90% of the NP core. This host matrix effectively shields the polar environment, reducing non-radiative energy loss and isolating TADF molecules from oxygen. Experiments have also demonstrated that TADF g-Odots easily enter cells, exhibit good absorption properties in living cells, and maintain long-term stability and traceability. Consequently, TADF g-Odots can potentially be used for long-term non-invasive cell tracking.

### *In vivo* imaging

*In vivo* imaging involves tracking and observing various biological processes at the cellular and molecular levels using imaging techniques. Non-invasive, real-time, and continuous dynamic monitoring of target molecules *in vivo* is crucial for studying important physiological processes and diseases in organisms. *In vivo* imaging techniques include visible light, ultrasound, X-ray, and isotope imaging.^89^^,^^90^^,^^91^^,^^92^^,^^93^ However, *in vivo* fluorescence imaging is often affected by absorption, scattering, and autofluorescence by the tissues of an organism.^94^ Organic TADF molecules offer advantages such as low production cost and biotoxicity, high luminescence efficiency, and a long fluorescence lifetime. Fluorescent probes designed based on organic TADF molecules can effectively eliminate interference from short-lived background fluorescence, significantly improving the signal-to-noise ratio and the quality of *in vivo* fluorescence imaging.

#### Mouse imaging

As model organisms, mice are small and have relatively transparent tissue structures, making them suitable for *in vivo* imaging. Mouse *in vivo* imaging provides real-time, non-invasive, and quantitative data and information, which is valuable for understanding biological processes, disease mechanisms, and drug effects.^95^^,^^96^^,^^97^^,^^98^ However, existing fluorescence imaging systems have limitations, such as a low image signal-to-noise ratio and limited penetration depth. NIR-II fluorescence imaging can be employed to achieve ideal imaging results. Compared to traditional visible light and NIR-I imaging, NIR-II fluorescence imaging offers a deeper penetration depth, higher signal-to-noise ratio, and better spatial resolution, making it more suitable for *in vivo* imaging and monitoring biological information in deep tissues.^11^^,^^99^^,^^100^^,^^101^^,^^102^

**Figure 7:**
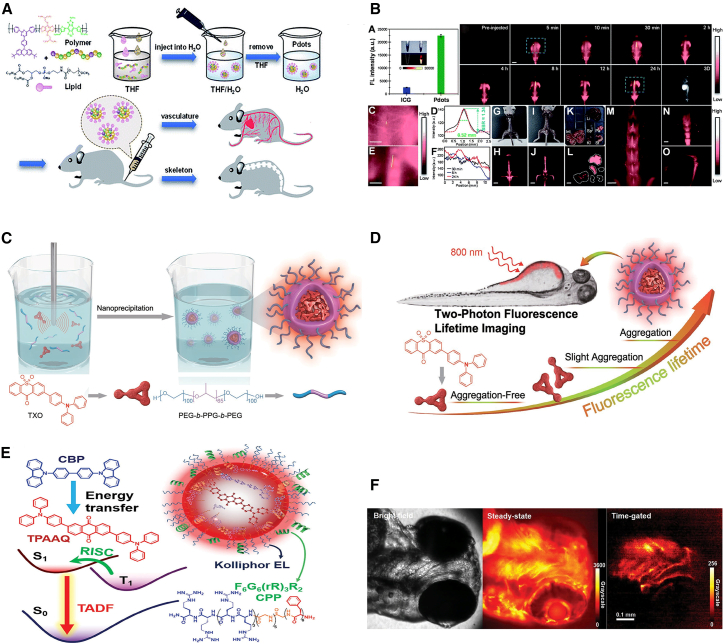
TADF material for live imaging (A) Schematic diagram representing the preparation of lipid encapsulated Pdots for *in vivo* vascular and bone imaging. (B) Real-time whole-body imaging of vascular structures and bones in BALB/c mice (*n* = 5) intravenously injected with BDT-SBOT2 Pdots in the prone position at certain time intervals from 5 min to 24 h. (A and B) Reproduced with permission from Chan et al.^55^ Copyright 2022, Royal Society of Chemistry. (C) Illustration of the preparation of TXO NPs via nanoprecipitation. (D) Schematic illustration for maximizing aggregation of organic fluorophores to prolong fluorescence lifetime for TP-FLIM. (C and D) Reproduced with permission from Fan et al.^109^ Copyright 2018, Wiley. (E) Schematic of TPAAQ/CBP/CPP NPs through host-guest assembling. (F) Bright-field (left), steady-state luminescence (middle), and time-gated luminescence (right) imaging of zebrafish after incubation with TPAAQ/CBP/CPP nanoparticles. (E and F) Reproduced with permission from Wu et al.^112^ Copyright 2023, Wiley.

Chan et al. synthesized TADF-containing conjugated polymers by copolymerizing TADF monomers with donor and acceptor segments through Stille coupling and then assembled semiconductor Pdots with NIR-II emission by co-precipitating the conjugated polymers with amphiphilic lipids.^55^ These Pdots demonstrated low cytotoxicity and excellent photostability, with emission maxima in aqueous solutions at 1,064–1,100 nm and fluorescence quantum yield of 0.40%–1.58% (Figure 7A). Among them, BDT-SBOT2 Pdots, with a higher fluorescence quantum yield, were intravenously injected at a concentration of 5 mg/mL (200 μL per mouse) for real-time imaging of the whole body and bones of mice. After tail vein injection of Pdots into live mice, whole-blood imaging was performed over time (Figure 7B). The Pdots were distributed throughout the body within 5 min and began accumulating in the excretory system and bones 2 h post-injection. The signal-to-background ratio for the spinal cord vessels was 1.34, and the spatial resolution was 0.52 mm. Studies have shown that bone-targeting efficiency is related to the degree of PEGylation and PEG modification of the probe. Since BDT-SBOT2 Pdots were covered with PEGylated DSPE phospholipids, these Pdots effectively targeted bones and are further used for imaging bone-related diseases and bone metastasis research.

#### Zebrafish imaging

As model vertebrates, zebrafish have high genetic and physiological similarities to humans and are widely used in developmental biology, genetics, and neuroscience research. During the embryonic and larval stages, zebrafish are entirely transparent, making them ideal for optical *in vivo* imaging. It is significant for long-term monitoring of changes in organ tissues and cells at different developmental stages.

Two-photon FLIM is based on two-photon excitation fluorescence imaging, which acquires sample information by measuring the lifetime of the fluorescent particles. This technique provides high spatial resolution and deep penetration capability, allowing high-resolution imaging in deep tissues without damaging cells and tissues, thus serving as a powerful tool for *in vivo* tissue imaging.^103^^,^^104^^,^^105^^,^^106^ The development of two-photon organic TADF probes overcomes the interference from the intrinsic fluorescence of biological samples and exhibits longer fluorescence lifetimes, making them novel imaging agents for FLIM.^107^^,^^108^ Fan et al. prepared organic semiconductor NPs (TXO NPs) with efficient two-photon absorption by encapsulating TADF materials based on thiophene-anthrone into an amphiphilic polymer (PEG-b-PPG-b-PEG) using nanoprecipitation (Figure 7C).^109^ TXO NPs exhibited TADF properties and AIE characteristics, maximizing TXO aggregation within the polymer matrix and achieving an ultra-long fluorescence lifetime (4.2 μs) that is inert to oxygen, making TXO NPs suitable for two-photon fluorescence lifetime imaging microscopy (TP-FLIM) in oxygen-rich biological environments (Figure 7D). TXO NPs demonstrated good water solubility and biocompatibility. Confocal fluorescence imaging showed that TXO NPs easily entered the cytoplasm of HepG2 cells and exhibited a strong red fluorescence. Additionally, their *in vivo* TP-FLIM capability was studied in zebrafish, using a concentration of 10 μg mL^−1^ after 4-h incubation, showing that the relatively long fluorescence lifetime of TXO NPs effectively eliminated short-lived autofluorescence interference, making them suitable for *in vivo* TP-FLIM.

Using the fluorescence lifetime as the output signal, TRFI technology, independent of the penetration depth of the light signal in biological tissues, can effectively eliminate scattering and short-lived autofluorescence interference from biological tissues. Thus, obtaining high-resolution imaging results in tissues of varying thicknesses. Therefore, TRFI holds great potential for multi-channel detection in biological organisms, providing insights into complex physiological processes and enabling precise quantitative analysis.^110^^,^^111^ Most TADF molecules generally have short luminescence lifetimes, typically ranging from hundreds of nanoseconds to a few microseconds, and low luminescence efficiency. Therefore, many hydrophobic TADF molecules must be encapsulated into NPs, limiting their application in real-time TRFI. Wu et al. designed highly efficient red TADF NPs with sub-millisecond luminescence lifetimes.^112^ They co-assembled host-guest molecules, using CBP and TPAAQ as host and guest molecules, respectively, and incorporated cell-penetrating peptides and amphiphilic compounds (Kolliphor EL) into NPs (Figure 7E). With low CBP doping concentrations, the NPs exhibited significant red TADF with luminescence lifetimes greater than 0.1 ms and a PLQY of up to 35%. The long luminescence lifetime and high efficiency of TADF enabled the first real-time time-gated fluorescence imaging in zebrafish incubated with NPs (Kolliphor EL: 15 μg mL^−1^, CBP: 10 μg mL^−1^, TPAAQ: 0.5 μg mL^−1^, CPP: 1.25 μg mL^−1^) for approximately 1 h (Figure 7F). Time-resolved imaging results using a chopper showed that most tissue autofluorescence was eliminated, and long-lifetime luminescence near the olfactory organs was clearly observed, with a delay time of approximately 0.1 ms. Compared to time-resolved imaging with CLSM, the acquisition time was shortened by two orders of magnitude, with a total exposure time of only 0.5 s per image, demonstrating the successful application of TADF NPs for real-time *in vivo* time-gated fluorescence imaging in zebrafish without background fluorescence.

In zebrafish live-imaging studies, both D-A structured TADF probes and aggregation-induced emission TADF (AIE-TADF) probes exhibit excellent imaging performance, each with distinct advantages based on application scenarios and structural design. Typical D-A-D structured probes, such as TPAAQ/CBP/CPP NPs, utilize low-concentration doping of the TPAAQ guest molecule into a CBP host matrix with cell-penetrating peptide (CPP) modification, achieving long delayed fluorescence lifetimes (>0.1 ms, up to 0.2 ms) and high quantum yield (35%). Operating in the red-light region (610–650 nm), they support real-time, highly sensitive dynamic imaging—such as respiratory activity—when combined with time-gated techniques, offering excellent background suppression. In contrast, AIE-TADF probes like TXO NPs combine AIE characteristics with TADF properties, delivering stable luminescence in highly aggregated states with a 4.2 μs lifetime. These probes offer superior two-photon absorption for deep-tissue TP-FLIM in the NIR window (760–800 nm), along with outstanding water solubility, oxygen tolerance, and long-term stability. Therefore, AIE-TADF probes are more suitable for prolonged, deep tissue imaging in stable environments, such as embryonic development tracking, whereas D-A probes, with their short lifetimes and rapid imaging capabilities, are better for sensitive real-time imaging of dynamic physiological processes. Future research should aim to improve the luminescence efficiency and aggregation stability of D-A structures, while AIE-TADF materials require strategies like energy gap modulation to extend fluorescence lifetimes and enable excitation within the NIR-II window, thereby enhancing their performance in complex physiological conditions.

## Conclusion and perspectives

As a novel class of organic luminescent materials, TADF materials offer several advantages over traditional fluorescent materials, including simpler synthesis methods, diverse structural designs, lower cost, reduced biological toxicity, and high luminescence efficiency. These properties have garnered significant attention in various fields, such as OLEDs, biological imaging, and photodynamic therapy. TADF materials efficiently utilize all excitons for luminescence without requiring precious metals, primarily through RISC, which converts triplet excitons to singlet excitons, producing DF. This unique luminescence process effectively eliminates the interference of short-lived fluorescence signals, providing clearer and more accurate signals for biological imaging and other applications.

This review summarized the recent progress in encapsulation strategies for organic TADF materials in biological imaging and their applications in targeted organelle imaging, live cell tracking, and *in vivo* imaging. Despite significant advancements, several challenges remain in the application of TADF materials for imaging.

### Oxygen sensitivity and biocompatibility issues

The inherent oxygen sensitivity and limited biocompatibility of TADF materials hinder their application in biological imaging. While various encapsulation methods have been explored to protect TADF materials from polar environments and improve their dispersion and stability in aqueous media, these methods often dilute probe concentrations, reduce binding to target molecules, and complicate their use. Therefore, novel, highly efficient TADF molecular structures and better encapsulation strategies are essential.

### Excitation wavelength and penetration depth issues

Many TADF probes are currently excited by short wavelengths, which can generate background fluorescence and may also damage cells and tissues. Furthermore, these probes tend to have shallow penetration depths, limiting their ability to image and treat deep tissues. Hence, developing TADF materials that can be excited by visible or near-infrared (NIR) light is crucial. Additionally, three-photon imaging, offering better tissue penetration, resolution, and reduced photodamage compared to two-photon fluorescence imaging, presents a promising avenue for the future development of TADF probes.

### Diversity of targeting probes

While significant advancements have been made in developing TADF probes targeting specific organelles, effective probes remain unavailable for certain critical organelles, such as the Golgi apparatus, nucleus, and peroxisomes. This limitation underscores the necessity for further exploration and design of organelle-specific TADF probes, which would expand the toolkit for dynamic imaging and intracellular research. Moreover, long-term cellular tracking utilizing TADF probes is still in its nascent stages. A deeper understanding of the photophysical properties of TADF materials will be crucial for developing probes suitable for extended observation within live-cell environments.

### Biocompatibility and safety studies

For TADF probes to be safely and effectively used in biological imaging and biomedical applications, high biocompatibility and safety are paramount. Comprehensive, long-term studies on the absorption, distribution, excretion, metabolism, and toxicity of TADF materials within biological systems are necessary to ensure their safe application in living organisms.

In summary, while TADF materials hold significant potential for biological imaging, substantial challenges remain. By refining the molecular design and encapsulation strategies, as well as enhancing the biocompatibility and stability of TADF materials, their application in biological imaging and other biomedical fields will continue to expand and improve, leading to even more promising prospects in the future.

### The trade-off between extended lifetime and phototoxicity

TADF probes provide significant advantages for bioimaging, primarily due to their extended delayed fluorescence lifetimes that enhance signal-to-noise ratios by suppressing autofluorescence. However, this benefit is intrinsically linked to the generation of ROS mediated by long-lived triplet excitons, which can induce phototoxicity. The severity of this phototoxic effect is modulated by factors including excitation wavelength, probe design, and subcellular localization, with notably higher risks of oxidative damage entailed by high-energy UV-blue light excitation (350–488 nm) compared to red/NIR light (>600 nm). To mitigate phototoxicity risks while retaining the fluorescence lifetime advantage, the following strategies are recommended: prioritizing red/NIR excitation to minimize ROS generation; optimizing triplet-state dynamics, for instance by adjusting ΔE_s1-t1_ to reduce intersystem crossing efficiency; implementing robust oxygen-shielding encapsulation; and adopting stringent imaging protocols, including limitation of blue-light exposure duration and utilization of time-gated detection. Future probe development should integrate these strategies to ensure long-term stability and reproducibility. Furthermore, establishing standardized assessment protocols—encompassing ROS quantification and longitudinal biodistribution studies—will be critical for defining safety thresholds and confirming the viability of TADF probes for minimally invasive *in vivo* imaging applications.

## Acknowledgments

This project was supported by Science and Technology Development Plan Project of Jilin Province (20240601090RC).

## Author contributions

Y.X., conceptualization, writing—original draft, visualization, and investigation. M.Z., writing—review & editing and data curation. X.L., writing—review & editing and validation. K.S., supervision, project administration, funding acquisition, and writing—review & editing. Z.T., supervision, funding acquisition, and writing—review & editing. All authors have read and agreed to the published version of the manuscript.

## Declaration of interests

The authors declare no conflicts of interest.
